# Self-calibrated optical thermometer based on luminescence from SrLu_2_O_4_:Bi^3+^,Eu^3+^ phosphors

**DOI:** 10.1039/c8ra06358c

**Published:** 2018-10-16

**Authors:** Xueyan Chen, Zhigang Zheng, Liming Teng, Rongfei Wei, Fangfang Hu, Hai Guo

**Affiliations:** Department of Physics, Zhejiang Normal University Jinhua Zhejiang 321004 China ghh@zjnu.cn

## Abstract

Bi^3+^,Eu^3+^ co-doped SrLu_2_O_4_ phosphors were synthesized by a solid state reaction method. Their structural, luminescent and temperature sensing properties have been systematically investigated. The color-tunable emissions from violet to red were detected with the increase of Eu^3+^ concentration. Relying on energy transfer process from Bi^3+^ to Eu^3+^ and thermal quenching behaviour, the fluorescence intensity ratio (FIR) presents excellent temperature sensing performance. The maximum absolute and relative sensitivities reach 1.10% K^−1^ and 0.87% K^−1^, respectively. These meaningful results indicate that SrLu_2_O_4_:Bi^3+^,Eu^3+^ is a promising material for optical temperature sensing.

## Abstract 2

Bi^3+^,Eu^3+^ co-doped SrLu_2_O_4_ phosphors were synthesized by a solid state reaction method.

## Graphical abstract

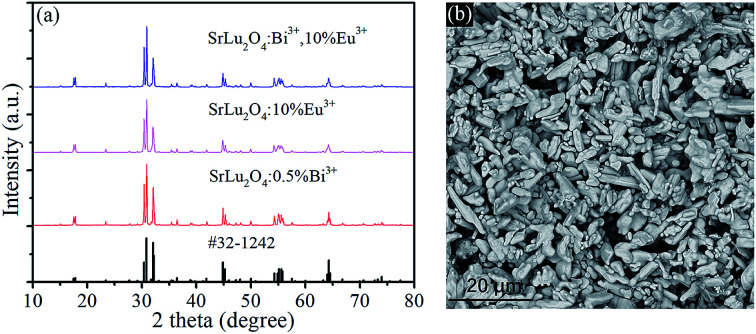

## Introduction

Recently, optical temperature sensors based on non-contact detection mode with rare earth (RE) ions doped luminescent materials have attracted extensive attention.^1–8^ Commonly, temperature sensing optical parameters, such as emission intensity, luminescent lifetime and the fluorescence intensity ratio (FIR) have been widely adopted for their non-contact operating mode.^5,9^ However, the measurement accuracy of temperature sensing strategy, which is based on the emission intensity, could be strongly affected by the external factors.^10^ Optical thermometry based on FIR technique, by contrast, can reduce the dependence of measurement conditions and have obvious advantages, such as no requirement for contact, high sensitivity, rapid response, as well as high spatial and temperature resolutions.^4^

Therefore, searching for a FIR temperature sensing material, whose spectra display two discriminable peaks with different temperature-dependent luminescent behaviours, is an important way to develop optical thermometry.^1^ In recent years, a series of FIR-based investigations have been systematically explored in order to search for high temperature sensitivity materials.^4,6,11^ The typical conventional researches focus on the thermally coupled energy levels (TCEL) of RE ions, the relative sensitivities of which are difficult to promote due to their inherent energy gap.^12,13^ Herein, a great many novel strategies and luminescent materials have been extensively investigated in order to promote relative sensitivity. According to the diverse thermal quenching behaviours of two intervalence charge transfer (IVCT) states, a novel thermometry strategy was proposed in 2016.^1^ Another typical strategy was based on the diversity in thermal behaviour of dual activators doped in separated matrixes.^14^ Other kind of thermometry strategy was based on the phonon assisted energy transfer between RE ions (*e.g.* Eu^3+^ and Tb^3+^) doped in metal–organic framework.^15^

It is well known that RE ions doped luminescent materials are plentiful luminescent resources due to their abundant energy levels.^16,17^ Eu^3+^ ion is a red activator originating from ^5^D_0_–^7^F_*J*_ (*J* = 0, 1, 2, 3, 4) transitions under UV light excitation.^17,18^ Bi^3+^ ion is another kind of activator, whose luminescence is attributed to the transition from 6s^2^ to 6s6p with broad absorption band in UV region.^19,20^ Bi^3+^ ion can emit various wavelength including ultraviolet, blue, green, yellow and even red bands in different hosts.^19^ These excellent luminescent properties make Bi^3+^ ion becomes a sensitizer to enhance Eu^3+^ emission in various hosts.^16–22^ Eu^3+^ doped SrLu_2_O_4_ and SrGd_2_O_4_ have been considered as promising red phosphors in solid state lighting devices.^23,24^ But energy transfer from Bi^3+^ to Eu^3+^ and temperature sensing property in SrLu_2_O_4_ have never been investigated.

In this work, a series of Bi^3+^,Eu^3+^ co-doped SrLu_2_O_4_ phosphors were successfully synthesized by a solid state reaction method. The structural, luminescent and temperature sensing properties were investigated in detail. With the increase of Eu^3+^ content in SrLu_2_O_4_:0.005Bi^3+^,*y*Eu^3+^, tunable emission including violet, pink and red can be observed owing to energy transfer from Bi^3+^ to Eu^3+^. Moreover, based on the opposite temperature dependence of Bi^3+^ and Eu^3+^ emission intensities, the temperature sensitivities reach high in the range from 315 to 543 K. More importantly, the main emission peak of Eu^3+^ and band of Bi^3+^ are well separated, providing an excellent signal discriminability for temperature sensing and detection.

## Experimental

A series of SrLu_1.9_O_4_:0.1Eu^3+^, SrLu_2−*x*_O_4_:*x*Bi^3+^ (*x* = 0, 0.25, 0.5, 1, 3%), and SrLu_2−0.005−*y*_O_4_:0.005Bi^3+^,*y*Eu^3+^ (*y* = 0, 0.5, 2.5, 6, 10 and 12.5%) samples were prepared by conventional solid state reaction method. In this paper, SrLu_1.9_O_4_:0.1Eu^3+^, SrLu_2−*x*_O_4_:*x*Bi^3+^, and SrLu_2−0.005−*y*_O_4_:0.005Bi^3+^,*y*Eu^3+^ are briefly labelled as SrLu_2_O_4_:10% Eu^3+^, SrLu_2_O_4_:*x*Bi^3+^, and SrLu_2_O_4_:Bi^3+^,*y*Eu^3+^, respectively. SrCO_3_ (A.R.), Bi_2_O_3_ (99.99%), Lu_2_O_3_ (99.99%) and Eu_2_O_3_ (99.99%) were used as starting materials. 2 wt% NH_4_Cl (A.R.) was used as flux. The raw materials were mixed in an appropriate molar ratio and thoroughly grounded for 30 min in an agate mortar. The powder mixtures were put into crucibles and sintered at 900 °C for 3 h in air. After repeated grinding, they were sintered at 1300 °C for 10 h in air. The resulted samples were cooled down to room temperature and pulverized. Then the final products were gained for further characterization.

The crystalline structure of phosphors were investigated by X-ray diffraction (XRD) on a Rigaku MiniFlex/600 X-ray diffraction apparatus (Tokyo, Japan) with CuKα (*λ* = 0.154056 nm) radiation at 40 kV and 15 mA in a step size of 0.02°. The morphologies of samples were recorded by scanning electron microscope (Phenom ProX desktop SEM). The excitation and emission spectra were performed on an Edinburgh FS5 spectrofluorometer equipped with a continuous wave 150 W Xe lamp. Temperature-dependent luminescent spectra were measured using FS5 spectrofluorometer equipped with a TCB1402C temperature controller (China). Lifetime measurement was acquired on an Edinburgh FLS920 spectrofluorometer equipped with a nanosecond flashlamp (nF900) as excitation source. All measurements were performed at room temperature except temperature-dependent spectra.

## Results and discussion

**Fig. 1:**
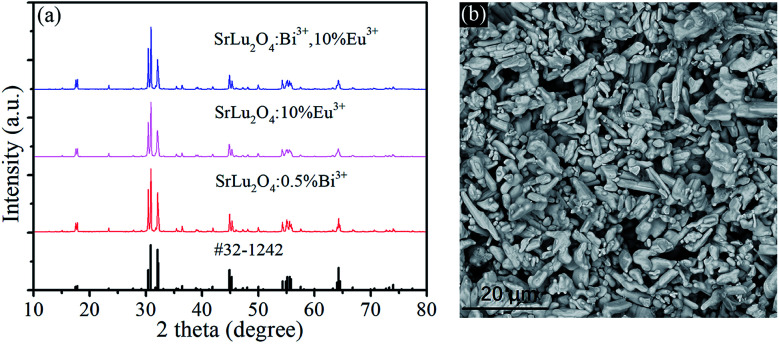
(a) XRD patterns of SrLu_2_O_4_:Bi^3+^,10% Eu^3+^, SrLu_2_O_4_:10% Eu^3+^, SrLu_2_O_4_:0.5% Bi^3+^, and the standard data of SrLu_2_O_4_ (JCPDS no. 32-1242) as a reference. (b) SEM micrograph of SrLu_2_O_4_:Bi^3+^,10% Eu^3+^.

Fig. 1(a) shows the XRD patterns of SrLu_2_O_4_:Bi^3+^,10% Eu^3+^, SrLu_2_O_4_:10% Eu^3+^ and SrLu_2_O_4_:0.5% Bi^3+^ samples with NH_4_Cl as flux, which can improve the crystallization, decrease the crystallization temperature and surface defect. Both Bi^3+^ (*r* = 1.03 Å, CN = 6) and Eu^3+^ (*r* = 0.947 Å, CN = 6) ions are suggested to occupy the sites of Lu^3+^ (*r* = 0.861 Å, CN = 6) ion due to their similar radii and valence states. No trace of impure phase is observed when Bi^3+^ or Eu^3+^ ions are doped into this lattice. SEM image displayed in Fig. 1(b) exhibits that the synthesized SrLu_2_O_4_ products are uniform with smooth facets, whose mean size is approximately 10 μm.

**Fig. 2:**
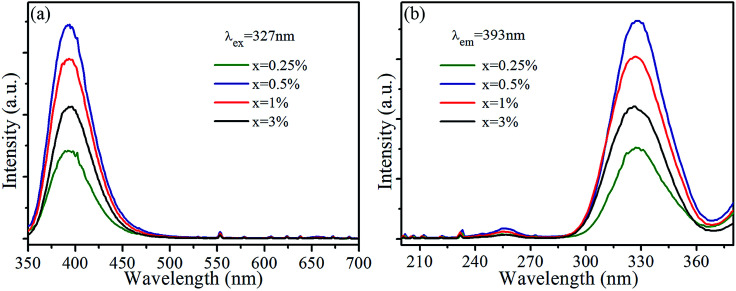
(a) Emission (*λ*_ex_ = 327 nm) and (b) excitation (*λ*_em_ = 393 nm) spectra of SrLu_2_O_4_:*x*Bi^3+^ (*x* = 0, 0.25, 0.5, 1, 3%) samples.

Room-temperature photoluminescence emission (*λ*_ex_ = 327 nm) and excitation (*λ*_em_ = 393 nm) spectra of SrLu_2_O_4_:*x*Bi^3+^ phosphors are presented in Fig. 2(a) and (b), respectively. Broadband blue emission from 350 to 510 nm with maximum at 393 nm is shown in emission spectra, which can be ascribed to the ^3^P_1_ → ^1^S_0_ transition of Bi^3+^.^21^ In excitation spectra monitored at 393 nm, there is an excitation band ranging from 300 to 360 nm centered at 327 nm which can be attributed to the spin-allowed ^1^S_0_ → ^3^P_1_ transition of Bi^3+^.^21^ With the increase of Bi^3+^ content, the emission and excitation intensities of Bi^3+^ increase sharply at first, reach the maximum at *x* = 0.5%, and then remarkably decrease when Bi^3+^ content is further increased due to concentration quenching.

**Fig. 3:**
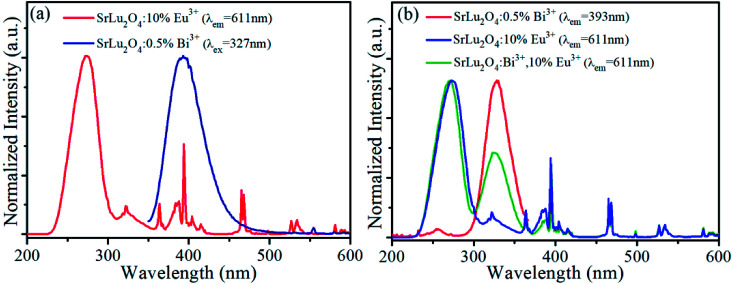
(a) Spectra overlap between excitation (*λ*_em_ = 611 nm) of SrLu_2_O_4_:10% Eu^3+^ and emission (*λ*_ex_ = 327 nm) of SrLu_2_O_4_:0.5% Bi^3+^. (b) Excitation (*λ*_em_ = 611 nm) spectra of SrLu_2_O_4_:10% Eu^3+^, SrLu_2_O_4_:Bi^3+^,10% Eu^3+^ and excitation (*λ*_em_ = 393 nm) spectra of SrLu_2_O_4_:0.5% Bi^3+^.

In order to explore energy transfer from Bi^3+^ to Eu^3+^ further, excitation spectra of SrLu_2_O_4_:10% Eu^3+^ (*λ*_em_ = 611 nm) and emission spectra of SrLu_2_O_4_:0.5% Bi^3+^ (*λ*_ex_ = 327 nm) are revealed in Fig. 3(a). In excitation spectra, there is a broadband from 230 to 305 nm centered at 274 nm, which can be ascribed to Eu^3+^–O^2−^ charge transfer transition.^25^ Due to ^7^F_0_ → ^5^D_4_, ^5^L_7_, ^5^L_6_, ^5^D_2_ and ^5^D_1_ transitions of Eu^3+^ ion, several weak excitation peaks at 322, 364, 394, 465, 527 nm can be observed.^19,21,25^ Meanwhile a broadband emission from 350 to 456 nm centered at 393 nm is presented in emission spectra ascribed to the ^3^P_1_ → ^1^S_0_ transition of Bi^3+^ ion. It is obvious that there is an overlap between emission spectra of Bi^3+^ and excitation spectra of Eu^3+^, indicating energy transfer process may occur from Bi^3+^ to Eu^3+^.

Normalized excitation spectra of SrLu_2_O_4_:10% Eu^3+^, SrLu_2_O_4_:Bi^3+^,10% Eu^3+^ and SrLu_2_O_4_:0.5% Bi^3+^ phosphors are displayed in Fig. 3(b). Excitation spectra of SrLu_2_O_4_:Bi^3+^,10% Eu^3+^ sample monitored Eu^3+^ emission (*λ*_em_ = 611 nm) ranging from 300 to 360 nm is almost similar with excitation spectra of SrLu_2_O_4_:0.5% Bi^3+^ sample monitored at 393 nm emission of Bi^3+^. These phenomena from Fig. 3 imply that a portion of emission intensity of Eu^3+^ comes from energy transfer process from Bi^3+^ to Eu^3+^.

On the other side, there is a band from 310 to 355 nm centered at 323 nm in excitation spectra of SrLu_2_O_4_:10% Eu^3+^. That means characteristic excitation wavelength of Bi^3+^ (*λ*_ex_ = 327 nm) can also excite Eu^3+^ ion directly. To be specific, a competitive absorption exists between Bi^3+^ and Eu^3+^ ions in SrLu_2_O_4_:Bi^3+^,10% Eu^3+^.

**Fig. 4:**
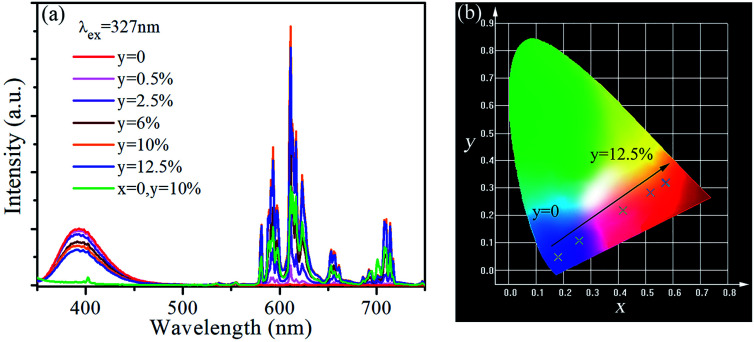
(a) Emission spectra of SrLu_2_O_4_:Bi^3+^,*y*Eu^3+^ (*y* = 0, 0.5, 2.5, 6, 10 and 12.5%) and SrLu_2_O_4_:10% Eu^3+^ under 327 nm excitation. (b) CIE chromaticity coordinates of SrLu_2_O_4_:Bi^3+^,*y*Eu^3+^ (*y* = 0, 0.5, 2.5, 6, 10 and 12.5%) (*λ*_ex_ = 327 nm).

The emission spectra of SrLu_2_O_4_:Bi^3+^,*y*Eu^3+^ (*y* = 0, 0.5, 2.5, 6, 10 and 12.5%) under 327 nm excitation are displayed in Fig. 4(a). As revealed in emission spectra, both emissions of Bi^3+^ and Eu^3+^ are observed. When the Eu^3+^ ion concentration increases, the red emission intensity increases rapidly and reaches the highest at 10%, after that the intensity decreases due to concentration quenching effect. However, the emission intensity from Bi^3+^ decreases slowly and monotonously with the increase of Eu^3+^ ion concentration. Therefore, conclusion can be drawn that the variation trend may owing to energy transfer from Bi^3+^ to Eu^3+^.

Fig. 4(b) gives the Commission International ed’Eclairage (CIE) chromaticity coordinates of SrLu_2_O_4_:Bi^3+^,*y*Eu^3+^ (*y* = 0, 0.5, 2.5, 6, 10 and 12.5%) phosphors under 327 nm excitation. The CIE chromaticity coordinates vary from (0.1805, 0.0478) to (0.5761, 0.3194) corresponding to *y* = 0–12.5%, locating in violet, pink and red regions. It is well-known that red and violet lights can promote photosynthesis effectively, which implies the SrLu_2_O_4_:Bi^3+^,Eu^3+^ phosphors may act as promising candidate to promote the photosynthesis of plant.^26–28^

The energy transfer efficiency from Bi^3+^ sensitizer to Eu^3+^ acceptor can be calculated by the following equation:^5,25,29^

**Fig. 5:**
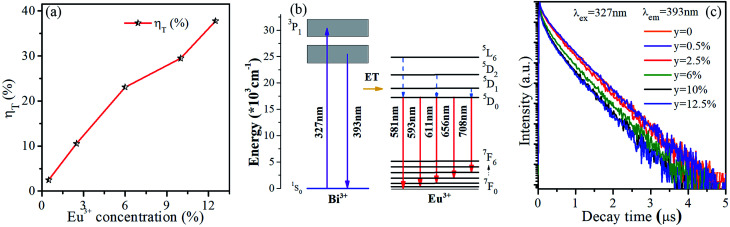
(a) Energy transfer efficiency as a function of Eu^3+^ concentration. (b) The schematic energy-level diagram of Bi^3+^ and Eu^3+^ ions and energy transfer process. (c) Luminescence decay curves of SrLu_2_O_4_:Bi^3+^,*y*Eu^3+^ (*y* = 0, 0.5, 2.5, 6, 10 and 12.5%) (*λ*_ex_ = 327 nm) monitored at 393 nm emission.

1 *η* = 1 − *I*_S_/*I*_S0_

where *η* is the energy transfer efficiency, *I*_S_ and *I*_S0_ are the integrated intensities of Bi^3+^ with and without Eu^3+^ ion in SrLu_2_O_4_ host, respectively. As displayed in Fig. 5(a), with the increase of Eu^3+^ ion concentration in SrLu_2_O_4_:Bi^3+^,*y*Eu^3+^ phosphors, the efficient energy transfer from Bi^3+^ to Eu^3+^ gradually increases and reaches maximum in the end concentration. However, the energy transfer efficiencies are all lower than 40% due to competitive absorption between Bi^3+^ and Eu^3+^ ions.

The luminescence decay curves of Bi^3+^ emission at 393 nm (*λ*_ex_ = 327 nm) in all phosphors were displayed in Fig. 5(c). The average lifetime *τ* can be evaluated by the following equation:^4,29^2
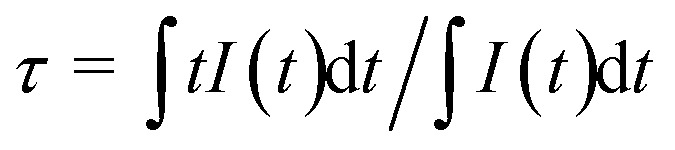
where *I*(*t*) is the emission intensity at time *t*. The lifetime *τ* of SrLu_2_O_4_:Bi^3+^,*y*Eu^3+^ (*y* = 0, 0.5, 2.5, 6, 10 and 12.5%) phosphors are 572, 569, 540, 494, 457 and 450 ns, respectively. It is obvious that the lifetime declines slightly with the increase of Eu^3+^ concentration, which illustrates the energy transfer from Bi^3+^ to Eu^3+^ is weak. Accordingly, more detailed investigation on energy transfer is inadaptable due to the competitive absorption between Bi^3+^ and Eu^3+^ ions.

**Fig. 6:**
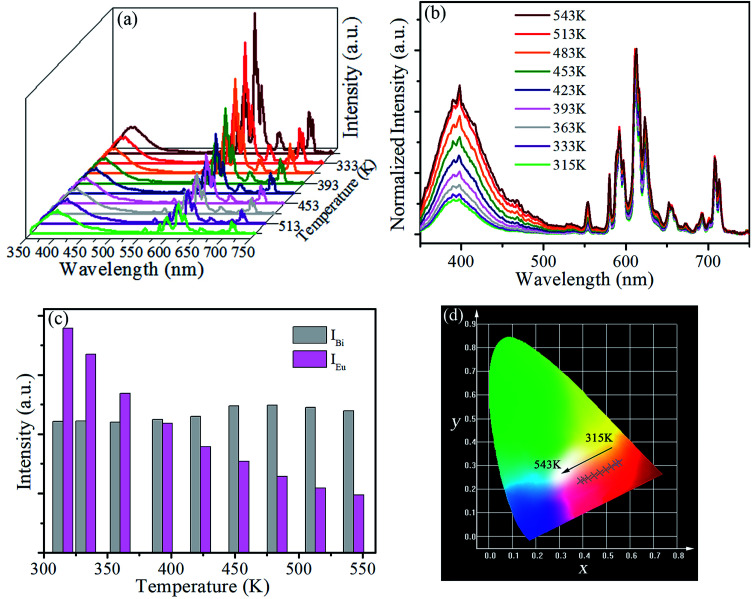
(a) Temperature-dependent emission spectra of SrLu_2_O_4_:Bi^3+^,10% Eu^3+^ sample recorded from 315 to 543 K. (b) The normalized emission spectra of SrLu_2_O_4_:Bi^3+^,10% Eu^3+^ sample. (c) Temperature-dependent emission intensities of Bi^3+^ and Eu^3+^ ions at various temperatures. (d) CIE chromaticity coordinates of the emission color at various temperatures.

Furthermore, temperature-dependent emission behaviour of SrLu_2_O_4_:Bi^3+^,10% Eu^3+^ under 327 nm excitation has been systemically investigated in order to explore possible temperature sensing material. As displayed in Fig. 6(a), the Eu^3+^ emission intensity decreases rapidly, while the Bi^3+^ emission intensity increases slightly with an increase of temperature from 315 to 543 K. To better exhibit the relative emission intensities variation, luminescent spectra in the research temperature range normalized to the 611 nm emission peak of Eu^3+^ is depicted in the Fig. 6(b).

The integrated intensities of Bi^3+^ and Eu^3+^, as shown in Fig. 6(c), exhibit that the emission intensity of Eu^3+^ decreases greatly from 315 to 543 K. In contrast, the emission intensity of Bi^3+^ presents a slight rise. Such opposite temperature-dependent luminescent behaviour was adopted to investigate for the temperature sensing performance of SrLu_2_O_4_:Bi^3+^,Eu^3+^ sample. Not surprisingly, the remarkable change in FIR (*I*_Bi_/*I*_Eu_) results in the shift of emission color from red to pink as the CIE diagram displayed in Fig. 6(d).

According to the theory proposed by Struck and Fonger, the relationship between temperature and emission intensity can be expressed as:^1^3
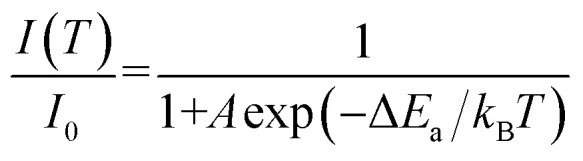
where *I*_0_ and *k*_B_ symbolize the emission intensity at 0 K and the Boltzmann constant, respectively. *A* is constant. The quenching activate energy representing the distance from the bottom of the excitation state to the intersection between the excitation and ground states is denoted by Δ*E*_a_.

To further assess the temperature sensing performance of the SrLu_2_O_4_:Bi^3+^,Eu^3+^ phosphor, the FIR (*I*_Bi_/*I*_Eu_) of Bi^3+^ to Eu^3+^ can be deduced from eqn (3) and expressed as follows:^30^4
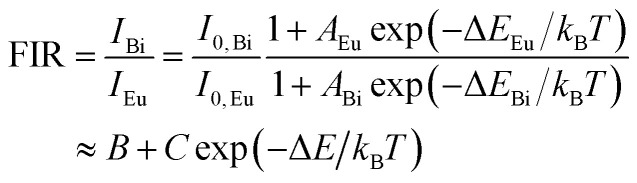
where *B*, *C* and Δ*E* are the parameters related to Bi^3+^ and Eu^3+^. The absolute sensitivity *S*_a_ and relative sensitivity *S*_r_ can be calculated and expressed as follows:^5^5
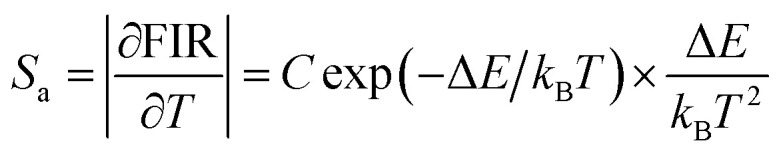
6
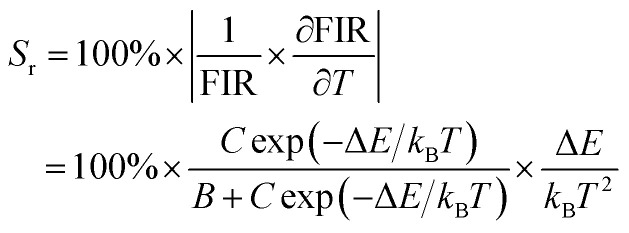


**Fig. 7:**
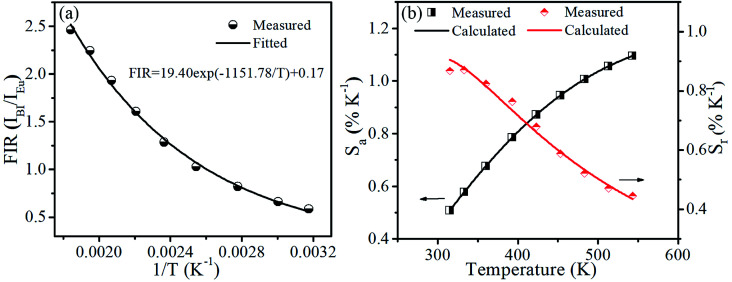
(a) Experimental measured and fitted plots of FIR (*I*_Bi_/*I*_Eu_) *versus* 1/*T*. (b) Absolute sensitivity *S*_a_ and relative sensitivity *S*_r_*versus* temperature.

The plots of temperature-dependent FIR can be fitted by eqn (4) from 315 to 543 K, as shown in Fig. 7(a). Consequently, the values of *B*, *C* and Δ*E* parameters in this fitting function can be determined to be 0.17, 19.40 and 1151.78 K, respectively.

Fig. 7(b) presents the *S*_a_ and *S*_r_, which were deduced by eqn (5) and (6), respectively. It is clear that absolute sensitivity *S*_a_ increases monotonously from 315 to 543 K, while the relative sensitivity *S*_r_ decreases with the rise of temperature. Noteworthily, SrLu_2_O_4_:Bi^3+^,10% Eu^3+^ exhibits high temperature sensitivities with the maximal values of *S*_a_ and *S*_r_ are 1.10% K^−1^ and 0.87% K^−1^, respectively. The obtained *S*_a-max_ is higher than most RE ions doped phosphor materials, as listed in Table 1.

**Table 1:** Optical parameters of several typical temperature sensing phosphor materials

Sensing materials	Temperature range (K)	*S*_a-max_	*T*_max_	Ref.
SrMoO_4_:Er^3+^,Yb^3+^	93–773	0.0128	480	31
NaLuF_4_:Yb^3+^,Er^3+^,Mn^2+^	295–525	0.01099	295	6
ZnWO_4_:Er^3+^,Yb^3+^	83–583	0.0099	583	2
YNbO_4_:Er^3+^	100–1600	0.0093	554	32
LuNbO_4_:Mo^6+^,Er^3+^	323–673	0.0069	—	33
Gd_2_O_3_:Er^3+^,Eu^3+^	300–443	0.0043	300	34
Gd_2_TiO_5_:Yb^3+^,Er^3+^	298–573	0.004076	565	35
Y_2_WO_6_:Tm^3+^,Yb^3+^	303–473	0.0034	303	36
BaTiO_3_:Er^3+^	300–450	0.0032	400	37
SrLu_2_O_4_:Bi^3+^,Eu^3+^	315–543	0.0110	543	This work

**Fig. 8:**
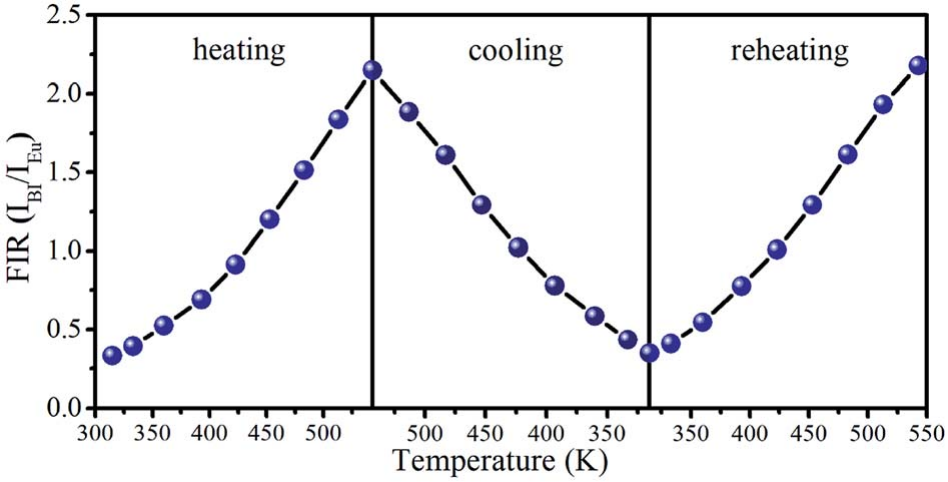
Temperature-dependent FIR (*I*_Bi_/*I*_Eu_) plots of in temperature cycling process from 315 to 543 K.

In order to evaluate reversibility, the temperature-recycle measurements are studied in SrLu_2_O_4_:Bi^3+^,10% Eu^3+^. As revealed in Fig. 8, the sample has an excellent repeatability of the temperature-dependent FIR. The above results demonstrate that SrLu_2_O_4_:Bi^3+^,Eu^3+^ phosphor is a promising material for optical temperature sensor.

## Conclusions

SrLu_2_O_4_ phosphors doped with different concentrations of Bi^3+^ and Eu^3+^ were successfully synthesized by solid state reaction method. The energy transfer from Bi^3+^ to Eu^3+^ was investigated by luminescent properties and decay curves. With the increase of the doped Eu^3+^ concentration, the color-tunable emissions from violet to red were detected. Owing to the competitive absorption between Bi^3+^ and Eu^3+^ ions, the energy transfer efficiency from Bi^3+^ to Eu^3+^ is about 37.8%. Temperature-dependent emission behaviour of SrLu_2_O_4_:Bi^3+^,10% Eu^3+^ under 327 nm excitation has been systemically investigated. Based on the opposite temperature dependence, the maximum absolute and relative sensitivities reach as high as 1.10% K^−1^ and 0.87% K^−1^, respectively. The excellent temperature sensing performance indicates SrLu_2_O_4_:Bi^3+^,10% Eu^3+^ is a promising candidate in the fields of optical temperature sensor.

## Conflicts of interest

The authors declare that there is no conflict of interests regarding the publication of this article.

## Supplementary Material
